# Chronic disease management improves survival but not hospital presentations: a target trial approach using linked data from the Australian Stroke Clinical Registry.

**DOI:** 10.23889/ijpds.v7i3.1885

**Published:** 2022-08-25

**Authors:** Nadine Andrew, David Ung, Muideen Olayia, Lachlan Dalli, Leonid Churilov, Dominique Cadilhac, Vijaya Sundararajan, Amanda Thrift, Natasha Lannin, Mark Nelson, Velandai Srikanth, Monique Kilkenny

**Affiliations:** 1 Peninsula Clinical School, Central Clinical School, Monash University; 2 Stroke and Ageing Research, Department of Medicine, School of Clinical Sciences at Monash Health, Monash University; 3 Florey Institute of Neuroscience and Mental Health; 4 Department of Public Health, School of Psychology and Public Health, La Trobe University; 5 Department of Neuroscience, Central Clinical School, Monash University; 6 University of Tasmania and Menzies Institute for Medical Research

## Objectives

Data linkage can provide sufficient breadth and size of data, to draw reliable estimates of effectiveness, at a population level using real-world data. We compared differences in survival and hospital presentations following stroke or transient ischaemic attack (TIA), based on whether a Medicare funded chronic disease management plan was claimed.

## Approach

A population-based, comparative effectiveness study of Victorian and Queensland Australian Stroke Clinical Registrants (January 2012-June 2015), using the emulated target trial approach, was performed. Chronic disease management items were identified from Medicare claims in the 6-18 months post-stroke (exposure period). Data on covariates for model adjustment were obtained from hospital, pharmacy and aged care datasets. Outcomes at 19-30 months post-stroke were determined using the national death registry and state hospital data. Cox regression, adjusted using propensity score methods with inverse probability treatment weights was used to determine the effect of receipt of chronic disease management claims on outcomes.

## Results

Of 28,775 AuSCR registrants, 27,435 (95.3%) were linked across the Medicare, pharmacy and hospital datasets. Following exclusions, 11,574 registrants from 42 hospitals (42% female, median age 70 years, 27% TIA) were eligible for the study. Overall, 45% of participants received chronic disease management during the exposure period. After propensity score weighting, there was excellent balance between groups across 35 baseline variables (standardised differences <0.1). Receipt of chronic disease management (vs non-receipt) was associated with a 30% reduced hazard of death (adjusted Hazard Ratio [aHR]: 0.70, 95%CI: 0.57, 0.87) but a 17% increase in hospital presentations (aHR: 1.17, 95%CI: 1.12, 1.23). Variation was observed between planned (aHR: 1.21, 95%CI: 1.10, 1.33) and unplanned (aHR: 1.15, 95%CI: 1.07, 1.23) presentations.

## Conclusion and Relevance

We provide an evaluation of the effectiveness of Medicare funded chronic disease management within “real world” healthcare provision, thereby demonstrating the value of linked population data in health services research. Further work is underway to examine causal mechanisms.

